# What do patients consulting in a free sexual health center know about HIV transmission and post-exposure prophylaxis?

**DOI:** 10.1186/s12889-021-10547-9

**Published:** 2021-03-12

**Authors:** Christelle Duteil, Elise de La Rochebrochard, Prescillia Piron, Christophe Segouin, Pénélope Troude

**Affiliations:** 1 Service de Santé Publique, Hôpitaux Lariboisière – Fernand-Widal, AP-HP Nord, F-75010 Paris, France; 2 Institut National d’Etudes Démographiques (Ined), F-93322 Aubervilliers, France; 3 Université Paris-Saclay, UVSQ, Inserm, CESP, F94807 Villejuif, France; 4 CeGIDD, Hôpitaux Lariboisière – Fernand-Widal, AP-HP Nord, F-75010 Paris, France

**Keywords:** HIV, Knowledge, False beliefs, Free HIV and sexually transmitted infection screening center, Post-exposure prophylaxis

## Abstract

**Background:**

Screening, condom use and post-exposure prophylaxis (PEP) are among existing HIV prevention strategies. However, efficient use of these strategies requires that patients have an adequate knowledge of HIV transmission routes and awareness of risk behaviors. This study aimed to assess knowledge about HIV transmission among patients who attended a free HIV and sexually transmitted infection (STI) screening center in Paris, France, and to explore the patient profiles associated with HIV-related knowledge.

**Methods:**

This observational cross-sectional study included 2002 patients who attended for STI testing from August 2017 through August 2018 and completed a self-administered electronic questionnaire. Based on incorrect answers regarding HIV transmission, two outcomes were assessed: lack of knowledge and false beliefs. Factors associated with these two outcomes were explored using univariate and multivariate logistic regressions.

**Results:**

Only 3.6% of patients did not know about HIV transmission through unprotected sexual intercourse and/or by sharing needles. More than one third of patients (36.4%) had at least one false belief, believing that HIV could be transmitted by sharing a drink (9.7%), kissing (17.6%) or using public toilets (27.5%). A low educational level and no previous HIV testing were associated in multivariate analyses with both lack of knowledge and false beliefs. Age and sexual orientation were also associated with false beliefs. Furthermore, 55.6% of patients did not know that post-exposure prophylaxis consists of taking emergency treatment as soon as possible after risky intercourse.

**Conclusions:**

Although the main HIV transmission routes are well known, false beliefs persist and knowledge regarding PEP needs to be improved. Prevention campaigns must focus on these themes which appear as a complementary strategy to pre-exposure prophylaxis to reduce HIV infection.

**Supplementary Information:**

The online version contains supplementary material available at 10.1186/s12889-021-10547-9.

## Background

Human immunodeficiency virus (HIV) remains a public health challenge worldwide. An estimated 37.9 million people were living with HIV and nearly 1.7 million people were newly infected in 2018 [1]. Almost two thirds of new HIV infections were observed in Africa, with 800,000 new infections in Eastern and Southern Africa in 2018. However, HIV also remained a public health issue in high-income countries, as 68,000 new infections were estimated in Western and Central Europe and in North America in 2018 [2]. With 6200 new infections, France is one of the Western countries most affected by HIV [3]. Despite facilitated access to HIV care, late-stage diagnosis represents a third of new HIV diagnoses in France and half of these new diagnoses concerned people who had never been tested before [4]. HIV prevention strategies that include screening, condom use, needle exchange programs, treatment of sexual transmitted infections and also treatment as prevention (TasP), post-exposure prophylaxis (PEP) and pre-exposure prophylaxis (PrEP) are needed to reduce the epidemic [5]. However, if they are to be effective, people must be aware of these strategies. For instance, a minimum of knowledge is needed for efficient use of PEP as it is an emergency treatment that must be taken as soon as possible and within 48-72 h (48 h in France) after potential exposure to HIV to reduce the risk of becoming seropositive. Use of PEP therefore requires knowledge of HIV transmission routes and awareness of risk behaviors.

Despite decades of public health information policies, knowledge about HIV may in fact have decreased, particularly among young people. According to the last national surveys in France, between 1994 and 2010 there has been a decrease in prevention practices, a decline in the level of HIV risk perception and an increase in erroneous beliefs regarding HIV transmission among young adults (18–29 years) [6]. Studies from other countries have underlined sexual risk behaviors and lack of HIV knowledge among young students, supporting the need for preventive and informative campaigns at earlier stages of adolescence [7–10]. Although most recent studies have focused on young people (college and university students), lack of HIV knowledge may also affect older people [11]. Some studies have also reported an association between HIV knowledge and factors such as sexual orientation, previous HIV testing or unprotected sexual intercourse [7, 12–16].

In France, screening centers for HIV and sexually transmitted infections (STIs) provide free and anonymous screening in order to reach socially disadvantaged and high-risk populations. Since 2016, the mission of these centers has expanded to address overall sexual health. Patients’ visits to screening centers provide opportunities to spread sexual health messages and to provide individual information about sexual risk behaviors [17]. HIV voluntary counseling and testing (HCT) recipients were significantly less likely to have unprotected sexual intercourse after than before HCT, or compared with participants who had not received HCT [17].

This study aimed to assess the level of knowledge regarding HIV transmission of patients who attended a free HIV and STI screening center and to explore the patient profiles associated with HIV knowledge.

## Methods

### Setting

This cross-sectional observational study was conducted in a free HIV and STI screening center in a Paris hospital (Fernand-Widal Hospital). The center offers tests for HIV, HBV, HCV, syphilis, chlamydia and gonorrhea. Since August 2017, every new patient who understands written French is invited by the reception agent to complete an optional and anonymous electronic self-administered questionnaire. Patients complete the questionnaire prior to medical consultation on a dedicated computer in a private area in the waiting room.

### Study population

From August 2017 through August 2018, 3589 patients visited the screening center for HIV or other STI testing. We excluded from the study population patients younger than 18 or older than 54 years (*n* = 223) and those who did not understand written French (*n* = 304). Of the 3062 eligible patients, 2002 completed the self-administered questionnaire and were included in the study.

### Data collection

Data were obtained from the consultation database and the self-administered questionnaire. The consultation database contained date of consultation, gender, year of birth, STIs tested, test results, and date of return for results. The self-administered questionnaire (see Additional file 1) included three sets of variables: sociodemographic data, sexual behaviors or practices, and knowledge or beliefs regarding HIV transmission routes and prevention. Firstly, it included sociodemographic data: year of birth, gender, nationality, work status, educational level and health insurance coverage. Secondly, it included sexual behaviors and practices: gender of sexual partner(s), previous HIV testing, and having at least one act of unprotected sexual intercourse with partner(s) of unknown HIV status since the last HIV testing or ever. Sexual orientation was defined as a four-category variable: men who have sex with men (MSM), men who have sex exclusively with women, women who have sex with women (WSW) and women who have sex exclusively with men. Finally, the questionnaire included true and false statements to assess the level of knowledge regarding HIV transmission and post-exposure prophylaxis. For each statement, patients had the option of choosing “yes”, “no”, or “don’t know”. Participants who answered “don’t know” were grouped with the incorrectly answers. Knowledge was assessed by two questions related to true HIV transmission routes (the correct answer was “yes”): having unprotected sexual intercourse and sharing used needles. False beliefs regarding HIV were assessed by three questions related to false HIV transmission routes (the correct answer was “no”): sharing a drink with an infected person, kissing an infected person, and using public toilets.

### Statistical analysis

First, sociodemographic data, sexual and prevention practices of the study population were described. We also detailed answers to statements regarding HIV transmission routes and PEP to fulfill our primary goal. Then, to fulfill our secondary goal, i.e. to explore factors associated with knowledge level, two outcomes were considered. First, a *lack of knowledge of HIV transmission routes* was defined as giving at least one incorrect answer to the two questions on true modes of HIV transmission (“unprotected sexual intercourse” and “sharing used needles”). Secondly, having *false beliefs on HIV transmission* was defined as giving at least one incorrect answer to the three questions on false HIV transmission routes (sharing a drink with an infected person, kissing an infected person, and using public toilets).

Factors associated with each of the two outcomes were studied using univariate and multivariate logistic regression models. Because of the small numbers of events, we adopted a parsimonious methodology for multivariate analyses. For each studied outcome, multivariate models included factors selected by a stepwise backward selection method with a threshold of 0.2 for removal from the model. Associations were considered as statistically significant when *p* < 0.05. Statistical analyses were performed using STATA/SE 13.1 (Stata Corporation, College Station, TX, USA).

## Results

### Description of the study population

Of the 3062 eligible patients, 2002 completed the questionnaire and were included in the present study (participation rate 65%). The characteristics of the study population are presented in Table 1 (*n* = 2002). Two thirds of patients were men (65.6%) and nearly half (45.5%) were 25–34 years old. Half of the study population was employed (52.3%), one third was in training (33.7%) and most patients had at least a high school diploma (91.0%). A minority declared they had no health insurance coverage (9.0%). Almost one third (30.1%) had had at least one act of unprotected sexual intercourse with a person of unknown HIV status. More than three quarters of the study population had already been tested for HIV (77.9%). Regarding screening results, 10.2% of patients had at least one positive result and the infection most frequently tested was chlamydia (see Additional file 2).

### Level of knowledge and false beliefs regarding HIV transmission and level of knowledge on post-exposure treatment

HIV knowledge is presented in Table 2. Only 1.2% of the study population [95% CI, 0.7 to 1.7] did not know that HIV transmission is possible through unprotected sexual intercourse and 3.3% [95% CI, 2.5 to 4.1] through sharing used needles. In summary, 3.6% [95% CI, 2.8 to 4.4] of the study population had a *lack of knowledge of HIV transmission routes* (i.e they had given an incorrect answer to at least one of the two items). Concerning false HIV transmission routes, 9.7% [95% CI, 8.4 to 11.0] believed that HIV transmission is possible by sharing a drink with an infected person, 17.6% [95% CI, 15.9 to 19.3] by kissing an infected person and 27.5% [95% CI, 25.5 to 29.4] by using public toilets. In summary, more than one third of the study population, 36.4% [95% CI, 34.3 to 38.5] had *false beliefs on HIV transmission* (i.e they had given an incorrect answer to at least one of the three items). Furthermore, 55.6% [95% CI, 53.4 to 57.7] of the study population did not know that post-exposure prophylaxis consists of taking emergency treatment as soon as possible after risky intercourse.

**Table 1:** Sociodemographic characteristics of the study population and sexual and prevention practices (*n* = 2002)

	n	%
**Age (years)**		
18–24	716	35.8
25–34	912	45.5
35–54	374	18.7
**Gender**		
Men	1314	65.6
Women	688	34.4
**French nationality**		
Yes	1752	87.5
No	250	12.5
**Activity status**		
Employed	1052	52.5
Student/Training	677	33.8
Unemployed	273	13.6
**Educational level**		
< High school diploma	161	8.0
High school diploma	456	22.8
> High school diploma	1385	69.2
**Health insurance coverage**		
Yes	1822	91.0
No	180	9.0
**Sexual orientation**		
Men who have sex exclusively with women	892	44.5
Men who have sex with men	422	21.1
Women who have sex exclusively with men	594	29.7
Women who have sex with women	94	4.7
**Previous HIV testing**		
None	443	22.1
Yes, one	490	24.5
Yes, several	1069	53.4
**At least one positive result**		
Yes	204	10.2
No	1798	89.8
**At least one act of unprotected sexual intercourse with a partner of unknown HIV status**		
Yes	603	30.1
No	1399	69.9

**Table 2:** Knowledge of HIV transmission and post-exposure prophylaxis (*n* = 2002)

	Correct answer	% Incorrect^a^ answer (n)
**HIV transmission is possible from …**		
unprotected sexual intercourse^b^	Yes	1.2 (24)
sharing used needles^b^	Yes	3.3 (66)
sharing a drink with an infected person ^c^	No	9.7 (195)
kissing an infected person^c^	No	17.6 (353)
using public toilets^c^	No	27.5 (550)
**Post-exposure prophylaxis (PEP) consists of …**		
taking emergency treatment as soon as possible after risky intercourse	Yes	55.6 (1113)

^a^Incorrect answers included “Don’t know”

^b^statement taken into account for the *Lack of knowledge of HIV transmission routes* outcome

^c^statement taken into account for the *False beliefs on HIV transmission* outcome

**Table 3:** Factors associated with HIV-related knowledge (n = 2002)

	Lack of knowledge of HIV transmission routes								False beliefs on HIV transmission							
	Univariate analysis					Multivariate analysis^a^			Univariate analysis					Multivariate analysis^a^		
	n/N	%	OR	95% CI	*p*	aOR	95% CI	*p*	n / N	%	OR	95% CI	*p*	aOR	95% CI	p
**Age (years)**					*0.910*								*< 0.001*			*< 0.001*
18–24	27 / 716	3.8	1.01	0.52–1.95		*–*	–	–	296 / 716	41.3	2.55	1.91–3.40		1.93	1.40–2.65	
25–34	31 / 912	3.4	0.90	0.48–1.72		*–*	–	–	352 / 912	38.6	2.27	1.72–3.00		2.08	1.56–2.78	
35–54	14 / 374	3.4	ref			*–*	–	–	81 / 374	21.7	ref			ref		
**French nationality**					*0.001*			*0.019*					*0.677*	–	–	*–*
Yes	54 /1752	3.1	ref			ref			635 / 1752	36.2	ref			–	–	*–*
No	18 / 250	7.2	2.44	1.40–4.23		2.06	1.13–3.76		94 / 250	37.6	1.06	0.81–1.39		–	–	*–*
**Activity Status**					*0.741*								*0.015*			
Employed	36 /1052	3.4	ref			–	–	*–*	352 / 1052	33.5	ref			–	–	*–*
Student/training	24 / 677	3.6	1.04	0.61–1.76		–	–	*–*	269 / 677	39.7	1.31	1.07–1.60		–	–	*–*
Unemployed	12 / 273	4.4	1.30	0.67–2.53		–	–	*–*	108 / 273	39.6	1.30	0.99–1.71		–	–	*–*
**Educational level**					*< 0.001*			*< 0.001*					*< 0.001*			*< 0.001*
< High school diploma	23 / 161	14.3	8.38	4.68–15.02		6.58	3.59–12.06		83 / 161	51.6	2.18	1.57–3.03		2.03	1.44–2.86	
High school diploma	22 / 456	4.8	2.55	1.44–4.52		1.94	1.07–3.52		192 / 456	42.1	1.49	1.20–1.85		1.30	1.02–1.65	
> High school diploma	27 /1385	2.0	ref			ref			454 / 1385	32.8	ref			ref		
**Health insurance coverage**					*< 0.001*			*0.002*					*0.008*			*0.110*
Yes	52 /1822	2.9	ref			ref			647 /1822	35.5	ref			ref		
No	20 / 180	11.1	4.25	2.48–7.3		2.53	1.39–4.59		82 / 180	45.6	1.52	1.12–2.07		1.30	0.94–1.79	
**Sexual orientation**					*0.405*	–	–	*–*					*< 0.001*			*0.006*
Men who have sex with men	12 / 422	2.8	ref			–	–	*–*	112 / 422	26.5	ref			ref		
Men who have sex exclusively with women	34 / 892	3.8	1.35	0.69–2.64		–	–	*–*	368 / 892	41.3	1.94	1.51–2.51		1.55	1.19–2.03	
Women who have sex exclusively with men	20 / 594	3.4	1.19	0.57–2.46		–	–	*–*	215 / 594	36.2	1.57	1.19–2.06		1.19	0.89–1.60	
Women who have sex with women	6 / 94	6.4	2.32	0.85–6.37		–	–	*–*	34 / 94	36.2	1.57	0.98–2.52		1.12	0.69–1.83	
**Previous HIV testing**					*< 0.001*			*0.002*					*< 0.001*			*0.002*
No	32 / 443	7.2	3.00	1.78–5.08		2.44	1.40–4.26		204 / 443	46.0	2.01	1.60–2.52		1.52	1.18–1.97	
Yes, one	13 / 490	2.7	1.05	0.54–2.06		0.92	0.46–1.83		206 / 490	42.0	1.70	1.37–2.13		1.40	1.11–1.78	
Yes, several	27 /1069	2.5	ref			ref			319 / 1069	29.8	ref			ref		
**At least one act of unprotected sexual intercourse with unknown HIV status partner**					*0.482*	–	–	*–*					*0.013*			*0.110*
Yes	19 / 603	3.2	0.83	0.49–1.41		–	–	*–*	244 / 603	40.5	1.28	1.05–1.56		1.18	0.96–1.45	
No	53 /1399	3.8	ref			–	–	*–*	485 / 1399	34.7	ref			ref		

Notes: *ref* reference category; ^a^The choice of the final multivariate model was based on the result of a stepwise backward selection with a 0.2 significance level for removal from the model

### Factors associated with lack of knowledge and with false beliefs

Table 3 presents univariate and multivariate regression models for each outcome studied: *1) lack of knowledge of HIV transmission routes* and *2) false beliefs on HIV transmission*.

1. In univariate analysis, non-French nationality, lower educational level, no health insurance coverage, and no previous HIV testing were associated with a *lack of knowledge of HIV transmission routes*. In multivariate analysis, all four factors remained significantly associated with a greater *lack of knowledge of HIV transmission routes:* non-French nationality (aOR, 2.06, 95% CI, 1.13 to 3.76), a low educational level (aOR, 6.58, 95% CI, 3.59 to 12.06) or a middle school level (aOR, 1.94, 95% CI, 1.07 to 3.52), no health insurance coverage (aOR, 2.53, 95% CI, 1.39 to 4.59), and no previous HIV testing (aOR, 2.44, 95% CI, 1.40 to 4.26).
2. In univariate analysis, *false beliefs on HIV transmission* were associated with age, activity status, educational level, health insurance coverage, sexual orientation and previous HIV testing. In multivariate analysis, six factors were retained by the stepwise selection procedure and four factors remained significantly associated with *false beliefs on HIV transmission:* age, educational level, sexual orientation and previous HIV testing. Patients under 35 years old were more likely to have false beliefs. The risk of false beliefs increased as the educational level decreased. Men who had sex exclusively with women were more likely to have *false beliefs on HIV transmission* (aOR, 1.55, 95% CI, 1.19 to 2.03) compared with MSM. No difference was found among women. No previous HIV testing (aOR, 1.52, 95% CI, 1.18 to 1.97) and only one previous HIV test (aOR, 1.40, 95% CI, 1.11 to 1.78) were significantly associated with *false beliefs on HIV transmission* in multivariate analysis.

## Discussion

Among patients consulting in a free screening center for HIV and STIs in Paris, only 3.6% had a *lack of knowledge of HIV transmission routes*, i.e. they did not know that HIV could be transmitted by unprotected sexual intercourse and/or by sharing used needles. However, three of 10 patients reported at least one act of unprotected sexual intercourse with a partner of unknown HIV status, and almost half did not know that post-exposure prophylaxis is an emergency treatment. Moreover, 36% of patients had *false beliefs on HIV transmission*. Socially disadvantaged patients and those with no previous HIV testing were more likely to have both insufficient knowledge and false beliefs regarding HIV transmission.

Our results observed in a population of patients consulting in a free screening center for HIV and STIs can be compared with the French general population only by using the most recent French national survey, the KABP France Group study (Knowledge, Attitudes, Beliefs and Practices), carried out in 2010 nearly one decade before our study [18]. The lack of knowledge about HIV transmission by sharing used needles was greater among patients of our free screening center than in the French general population in 2010. A few more recent studies have reported in detail on HIV-related knowledge [12, 19]. For instance, a Serbian study conducted in 2013–2014 among 1017 students reported that 2.7% of students did not know that HIV transmission is possible via sexual intercourse and 8.7% via intravenous drug equipment [12]. These studies reported higher levels of unawareness than ours, but comparison is limited due to the difference in settings. Taken together, these studies highlight the need for better targeting of prevention and education campaigns [20]. The spread of efficient treatments has changed the social representations of HIV/AIDS from ‘deadly’ to ‘chronic’ [21]. Change in representations may have reduced the fear of HIV and the attention paid to prevention campaigns.

More than one third of our study population had *false beliefs on HIV transmission*. The main false belief was that HIV transmission is possible by using public toilets (27.5% [95% CI, 25.5 to 29.4]). This false belief may have increased over the last decade in France, as this proportion was much lower in the 2010 French survey (16.8% [95% CI, 15.9 to 17.7]) [22]. Transmission of HIV using public toilets seems to be a common false belief that is largely held worldwide, as it has also been reported in Poland in a national opinion survey among adults (23%) [23], in Egypt among dental students (38%) [24], and in Korea among adolescents aged 16–18 years (40.5%) [25]. In our study, 17.6% also had false beliefs about HIV transmission through kissing an infected person and 9.7% through sharing a drink. These false beliefs are also observed in other populations, sometimes at a higher level [7, 26]. A study in the Kingdom of Saudi Arabia among young medical students showed that 52% thought HIV transmission was possible through a deep kiss and 18.6% through sharing a glass of water [26]. An American study among Hispanic men on college campuses observed that 41% thought HIV transmission was possible by kissing and 22% by sharing a glass [7]. The same false beliefs thus seem to be widely shared among populations of very different social and cultural backgrounds worldwide, even if the levels of these false beliefs are highly variable from one population to another.

False beliefs about HIV could be perceived as a less acute issue than lack of knowledge of HIV transmission routes. Indeed, false beliefs do not lead to HIV infection. However, such false beliefs may lead to discrimination and negative attitudes towards people living with HIV, for example avoidance of such persons [19]. Therefore, false beliefs should be considered as an important issue that needs to be addressed, especially among young people [20]. In a French survey conducted in 2019 in young people 15–24 years old, 23% felt poorly informed, compared with only 11% in 2009 [27]. Prevention and education campaigns should focus more specifically on young people [20].

Patients who had one or several previous HIV screening tests were less likely to have *lack of knowledge of HIV transmission routes* and *false beliefs on HIV transmission*. Some previous studies have reported an association between HIV knowledge and previous HIV testing [14, 28]. Participants who had undergone HIV testing were more likely to have a higher HIV knowledge score and a lower stigma score towards persons living with HIV than those who had not tested for HIV [7, 28]. A cross-sectional Chinese study among rural migrants showed that women with little HIV knowledge and low awareness regarding HIV risk were less willing to use voluntary counseling and testing [14]. An American study among adolescent MSM reported that those who had correct HIV knowledge were more likely to be tested for HIV [29]. Furthermore, 75.4% of MSM tested had previously spoken to a doctor about HIV testing compared with only 10.8% of those who had not had such conversations. On the other hand, a study conducted among students (18–24 years) in Tbilisi, Georgia, found that having had a HIV test was associated with not having a stigmatizing attitude towards HIV [30]. It is therefore not easy to interpret such an association between HIV knowledge and previous HIV testing. Possibly, patients who are more aware about HIV/STI may be more likely to come for STI screening, or screening may have been an opportunity to improve their knowledge. More studies, in particular prospective studies, are needed to clarify the relationship between HIV knowledge and HIV testing.

In our study, patients with a low educational level were significantly more likely to have *lack of knowledge of HIV transmission routes* and *false beliefs on HIV transmission*. This patient profile with low educational level has already been reported to be associated with a low level of HIV knowledge in previous studies [28, 31]. A Bangladeshi study among 12,593 married women found that a higher educational level was a major factor influencing a high score of HIV knowledge and awareness. Furthermore, wealth index and access to mass media were also associated with HIV knowledge and awareness [31]. However, these studies were conducted in developing countries, limiting comparison with our results.

In our study, sexual orientation was significantly associated with *false beliefs on HIV transmission* but not with *lack of knowledge of HIV transmission routes*. MSM had significantly less *false beliefs on HIV transmission* than men who have sex exclusively with women. This could be due to better targeted outreach and information campaigns to at-risk populations by community-based organizations [32]. The absence of a significant association between knowledge about HIV transmission and sexual orientation may be due to a lack of statistical power (only 72 patients had a *lack of knowledge of HIV transmission routes*). However, previous studies reported mixed results regarding an association between sexual orientation and HIV knowledge. Some studies found that MSM were aware of HIV transmission risks [15], but HIV knowledge may vary depending on how MSM self-identify [16]. Furthermore, a study conducted among Thai students reported that being homosexual or bisexual was associated with false perception of low HIV risk [33].

Despite a high level of knowledge regarding HIV transmission routes, our study revealed the need to improve knowledge about post-exposure prophylaxis (PEP). Only 44% of our study population knew that PEP is an emergency treatment to prevent HIV infection, although PEP has existed in France for more than 20 years (since 1998). Unawareness of PEP could lead to missed opportunities to avoid HIV infection. This lack of knowledge about PEP was reported in previous studies with variable levels of awareness according to the settings and populations. A study conducted among 400 participants who attended a clinic in Jerusalem for HIV testing reported lower awareness of PEP, with only 24% of participants being aware that the time window for effective PEP is 72 h [34]. In a South African study of 169 medical university students, only 28% reported that PEP can be used to prevent HIV infection [35]. Conversely, an American survey of 529 respondents in 2016–2017 reported a higher level of awareness, with 59% of participants being aware of PEP [32]. This American study pointed out significant differences of awareness among the three key populations studied, non-white young MSM, transgender women and non-white cisgender women, and reported a very high level of awareness among MSM (80%). The possibility of pre-exposure prophylaxis (PrEP) to prevent HIV transmission should not mask the role of PEP. For patients who are not candidates for PrEP due to infrequent HIV exposure or a lack of anticipation of sexual risk behavior, PEP remains an effective prevention tool to reduce HIV infections.

### Limitations and strengths of the study

This was a large study with a population of 2002 patients and a high questionnaire participation rate of 65%. It has, however, some limitations. Firstly, the questionnaire was only available in French and on a computer. The eligible population thus included only French-speaking patients, which could create a selection bias due to the language barrier. Moreover, the questionnaire was self-administered on a computer and it cannot be excluded that some patients did not feel comfortable with computer tools. This hypothesis is coherent with the slightly older age of non-participants in this study who may be less skilled with computers (mean age was 33.2 for non-participants vs 28.6 for participants) and the significant association between age and participation (*p* < 0.001). However, sexual behaviors and practices are sensitive data that are difficult to collect and self-reported data by computer could be a more efficient tool than face-to-face contact in order to reduce social desirability bias and improve data quality [36].

Secondly, the small number of patients who had a *lack of knowledge of HIV transmission routes* (*n* = 72) is an encouraging result, but restricted the study’s statistical power to explore associated factors in multivariate analysis. Lastly, the generalizability of the findings may be limited by the single-center design of our study. It would be interesting to carry out a similar study in other free HIV and STI screening centers in France.

## Conclusions

This study found that people who attended a free HIV and STI screening center were well aware of the main HIV transmission routes. However, false beliefs persist and could generate stigma towards people living with HIV. Our study highlights the fact that more than half of patients had insufficient knowledge about PEP. Prevention messages must focus on PEP and its availability should be facilitated. In addition to conventional PEP, an on-demand PEP called post-exposure prophylaxis-in-pocket (PIP) may be a useful strategy to prevent HIV [37, 38]. In order to reduce the HIV epidemic, people must know and use the combination of prevention tools that are available. Our results suggest that there is a need to improve awareness of HIV testing and prevention strategies and also to improve HIV-related knowledge, particularly among disadvantaged people. In addition to national prevention and information campaigns, free HIV and STI screening centers have a role to play in promoting sexual health, as screening may be a valuable opportunity to upgrade patients’ knowledge of HIV prevention tools and transmission routes.

## Supplementary Information

**Additional file 1.** Self-administered questionnaire (English language version).

**Additional file 2. **Prevalence of sexually transmitted infections (STIs) tested (*n* = 2002).

## Abbreviations

CI: Confidence interval
HIV: Human immunodeficiency virus
MSM: Men who have sex with men
PEP: Post-exposure prophylaxis
PrEP: Pre-exposure prophylaxis
STI: Sexually transmitted infection
