# Supplementary material for: What do patients consulting in a free sexual health center know about HIV transmission and post-exposure prophylaxis?
Source: BMC Public Health. 2021 Mar 12;21:494. doi: 10.1186/s12889-021-10547-9 (PMC7953800; doi:10.1186/s12889-021-10547-9)
Supplement: Supplementary file 1 — Additional file 1. Self-administered questionnaire (English language version). [file 12889_2021_10547_MOESM1_ESM.docx]

**Supplementary information**

**Additional file 1** Self-administered questionnaire (English language version)

| **Part 1 Your visit to the sexual health center** |
| --- |
| - 1. Anonymous number: _ _ _ _ _ _   *This 6-digit number is shown on the flyer you have been given.* |
| - 1. Year of birth: _ _ _ _ |
| 1-3 Have you ever had an HIV test?  (1) Yes, one  (2) Yes, several  (3) No  (4) Prefer not to answer |
| 1-4 Have you had anal or vaginal sexual intercourse without using a condom since your last test (HIV/STI), or ever in your life if you have never been tested?  (1) Yes  (2) No  (3) Prefer not to answer |
| 1-5 What was (were) the HIV status of your sexual partner(s)? (several possible responses)  (1) You knew they were HIV negative  (2) You thought they were HIV negative  (3) You did not know what their status was  (4) You didn't know they were HIV positive  (5) You knew they were HIV positive  (6) Prefer not to answer |
| **Part 2 Your personal situation** |
| 2-1 What is your gender?  (1) Male  (2) Female  (3) Transgender |
| 2-2 What is your nationality? (several possible responses)  (1) French  (2) Other: ________________________ |
| 2-3 What is your employment situation?  (1) Employed  (2) Unemployed  (3) Homemaker  (4) Student/in training  (5) Other (please state): _________________ |
| 2-4 What is your highest educational diploma?  (1) No educational qualification  (2) CAP/BEPC (vocational diplomas)  (3) BEP (vocational diploma)  (4) High school degree (vocational diploma)  (5) High school degree  (6) Diploma of Higher Education  (7) University degree or above  (8) Other (please state): _____ |
| 2-5 Do you have universal health insurance (PUMA, formerly called CMU)?  (1) Yes  (2) No  (3) Don’t know |
| 2-6 Do you have social security or other health insurance?  (1) Yes  (2) No  (3) Don’t know |
| 2-7 Do you have state medical aid (AME)?  (1) Yes  (2) No  (3) Don’t know |
| 2-8 Do you have complementary health insurance?  (1) Yes, CMUc (complementary universal health insurance)  (2) Yes, top-up or private insurance  (3) Yes, but don't know which one  (4) No  (5) Don’t know |
| **Part 3 Sexual life and prevention**  *We now ask you to answer some questions about your sex life and the prevention of sexually transmitted infections.*  *-> If you wish, you can talk about this with the doctor who will see you in consultation.* |
| 3-1 During your life, you have had sexual intercourse…  (1) Only with people of the opposite sex  (2) Only with people of the same sex  (3) With people of both sexes  (4) Never had sexual intercourse  (5) Prefer not to answer |
| 3-2 In the last 12 months, how many male sexual partners have you had?  Number: ____________ |
| 3-3 In the last 12 months, how many female sexual partners have you had?  Number: ____________ |
| 3-4 In the last 12 months, how many transgender MtF sexual partners have you had?  Number: ____________ |
| 3-4 In the last 12 months, how many transgender FtM sexual partners have you had?  Number: ____________ |
| **Part 4 HIV-related knowledge** |
| 4-1 Do you think HIV transmission is possible or not in each of the following situations?  (1) During unprotected sexual intercourse (yes/ no/ don’t know)  (2) Using public toilets (yes/ no/ don’t know)  (3) Sharing a drink with an infected person (yes/ no/ don’t know)  (4) Kissing an infected person (yes/ no/ don’t know)  (5) Sharing used needles (yes/ no/ don’t know) |
| 4-2 Have you ever heard about an emergency treatment that can be taken shortly after risky intercourse to reduce risk of HIV infection?  (1) Yes  (2) Possibly  (3) No  (4) Don’t know |
| 4-3 Do you know how soon you have to take this treatment after risky intercourse?  (1) I haven’t heard about it  (2) As soon as possible  (3) You have to wait about a week  (4) You have to wait a few weeks but less than three months  (5) You have to wait three to four months  (6) You have to wait even longer  (7) Don’t know |
