# Supplementary material for: What do patients consulting in a free sexual health center know about HIV transmission and post-exposure prophylaxis?
Source: BMC Public Health. 2021 Mar 12;21:494. doi: 10.1186/s12889-021-10547-9 (PMC7953800; doi:10.1186/s12889-021-10547-9)
Supplement: Supplementary file 2 — Additional file 2. Prevalence of sexually transmitted infections (STIs) tested (n = 2002). [file 12889_2021_10547_MOESM2_ESM.docx]

**Supplementary information**

**Additional file** **2** Prevalence of sexually transmitted infections (STIs) tested (n=2002)

| STI | n tested | n positive | % of positivity |
| --- | --- | --- | --- |
| HIV | 1946 | 6 | 0.3 |
| HBV | 1633 | 8 | 0.5 |
| HCV | 1324 | 5 | 0.4 |
| Syphilis | 1344 | 21 | 1.6 |
| Chlamydia | 1794 | 119 | 6.6 |
| Gonorrhea | 1794 | 69 | 3.9 |
| *At least one positive result* | *2002* | *204* | *10.2* |
